# Effect of Dural-Puncture Epidural vs Standard Epidural for Epidural Extension on Onset Time of Surgical Anesthesia in Elective Cesarean Delivery: A Randomized Clinical Trial

**DOI:** 10.1001/jamanetworkopen.2023.26710

**Published:** 2023-08-01

**Authors:** Nadir Sharawi, Matthew Williams, Waseem Athar, Caroline Martinello, Kyle Stoner, Cameron Taylor, Nan Guo, Pervez Sultan, Jill M. Mhyre

**Affiliations:** 1 Department of Anesthesiology, University of Arkansas for Medical Sciences, Little Rock; 2 Department of Anesthesiology, Duke University School of Medicine, Durham, North Carolina; 3 Department of Anesthesiology, Perioperative, and Pain Medicine, Stanford University School of Medicine, Stanford, California

## Abstract

**Importance:**

Dural-puncture epidural (DPE) and standard epidural are common modes of neuraxial labor analgesia. Little is known about conversion of DPE-initiated labor analgesia to surgical anesthesia for cesarean delivery.

**Objective:**

To determine whether DPE provides a faster onset and better-quality block compared with the standard epidural technique for cesarean delivery.

**Design, Setting, and Participants:**

This double-blind, randomized clinical trial was conducted between April 2019 and October 2022 at a tertiary care university hospital (University of Arkansas for Medical Sciences). Participants included women aged 18 years and older undergoing scheduled cesarean delivery with a singleton pregnancy.

**Interventions:**

Participants were randomized to receive DPE or standard epidural in the labor and delivery room. A T10 sensory block was achieved and maintained using a low concentration of bupivacaine with fentanyl through the epidural catheter until the time of surgery. Epidural extension anesthesia was initiated in the operating room.

**Main Outcomes and Measures:**

The primary outcome was the time taken from chloroprocaine administration to surgical anesthesia (T6 sensory block). The secondary outcome was the quality of epidural anesthesia, as defined by a composite of the following factors: (1) failure to achieve a T10 bilateral block preoperatively in the delivery room, (2) failure to achieve a surgical block at T6 within 15 minutes of chloroprocaine administration, (3) requirement for intraoperative analgesia, (4) repeat neuraxial procedure, and (5) conversion to general anesthesia.

**Results:**

Among 140 women (mean [SD] age, 30.1 [5.2] years), 70 were randomized to the DPE group, and 70 were randomized to the standard epidural group. The DPE group had a faster onset time to surgical anesthesia compared with the standard epidural group (median [IQR], 422 [290-546] seconds vs 655 [437-926] seconds; median [IQR] difference, 233 [104-369] seconds). The composite rates of lower quality anesthesia were 15.7% (11 of 70 women) in the DPE group and 36.3% (24 of 66 women) in the standard epidural group (odds ratio, 0.33; 95% CI, 0.14-0.74; *P* = .007).

**Conclusions and Relevance:**

Anesthesia initiated following a DPE technique resulted in faster onset and improved block quality during epidural extension compared with initiation with a standard epidural technique. Further studies are needed to confirm these findings in the setting of intrapartum cesarean delivery.

**Trial Registration:**

ClinicalTrials.gov Identifier: NCT03915574

## Key Points

**Question:**

Is dural-puncture epidural (DPE) superior to standard epidural when used for conversion to surgical anesthesia in patients undergoing elective cesarean delivery?

**Findings:**

In this randomized clinical trial of 140 participants, the onset time to surgical anesthesia was faster in the DPE group compared with the standard epidural group (422 vs 655 seconds).

**Meaning:**

These results suggest that DPE epidural extension anesthesia is superior to standard epidural, but these findings need to be confirmed in women requiring intrapartum cesarean delivery.

## Teaser

This randomized clinical trial examines the effect of dural-puncture epidural vs standard epidural for epidural extension on onset time of surgical anesthesia in individuals undergoing elective cesarean delivery.

## Introduction

Neuraxial analgesia is considered the criterion standard for labor and delivery, with an estimated 71% of parturients in the US receiving some form of neuraxial analgesia.^1^ Epidural and combined spinal-epidural are well established techniques for labor analgesia.^2^ Although dural-puncture epidural (DPE) is not necessarily a new technique, it has gained popularity and is used by some practitioners to initiate labor analgesia. Numerous studies^3,4,5,6^ have assessed block quality and adverse events between these neuraxial analgesia techniques in laboring patients. However, there are limited data comparing standard and DPE techniques when used to provide surgical anesthesia for cesarean delivery.

The DPE technique is similar to the combined spinal-epidural technique but without the injection of intrathecal drugs. First, the epidural space is identified with an epidural needle, and then a spinal needle is inserted through the epidural needle to puncture the dura. After confirmation of clear cerebrospinal fluid, the spinal needle is then removed, and the epidural catheter is inserted to initiate analgesia. The hypothesized advantage of the DPE over the standard epidural technique is improved analgesia secondary to epidural midline placement through the confirmation of cerebrospinal fluid flow through the spinal needle. Dural puncture with a DPE may also facilitate translocation of epidural medications into the intrathecal space to improve analgesia.^4,7^ However, in patients undergoing cesarean delivery under epidural extension anesthesia, the effects of DPE on surgical anesthesia block onset and reliability are unknown.

In view of the increasing use of DPE and its proposed advantages for labor analgesia, we conducted a double-blind, randomized clinical trial (RCT) to determine whether the DPE technique is superior to the standard epidural when used for epidural extension anesthesia in parturients undergoing elective cesarean delivery. We hypothesized that DPE would provide faster onset of surgical anesthesia compared with the standard epidural technique. We also sought to determine whether DPE would improve the overall quality and reliability of epidural anesthesia.

## Methods

This single-center, double-blind, RCT was approved by the institutional review board at the University of Arkansas for Medical Sciences and was conducted between April 2019 and October 2022. This article adheres to the applicable Consolidated Standards of Reporting Trials (CONSORT) reporting guidelines. The trial protocol and statistical analysis plan are provided in Supplement 1.

The authors recognize that there is a gender spectrum; however, all participants identified as women in this study, so we use this term throughout. We enrolled women with singleton pregnancies and American Society of Anesthesiologists physical status II or III who were scheduled for elective cesarean delivery. Participants were excluded if they were younger than 18 years, weighed more than 120 kg, were less than 150 cm tall, were unable to understand English, and had contraindications to neuraxial anesthesia, previous back surgery or scoliosis, severe fetal anomalies, or allergy to any study medications. Race and ethnicity were self-reported by the participants (Black, White, or other, which includes Asian, Hispanic ethnicity, and any other race or ethnicity not otherwise specified) and were included to assess the representativeness of the sample being studied.

After providing written informed consent, participants were randomly assigned to receive either standard epidural or DPE. A computer-generated, 1:1 ratio randomization schedule with blocks of 10 was created by a research coordinator not involved in the study. Allocation assignments remained concealed using sequentially numbered, opaque, sealed envelopes. Before scheduled cesarean delivery, the proceduralist opened the envelope containing the group allocation. Epidural procedures were performed by the attending anesthesiologist or by residents under direct supervision of the attending anesthesiologist. The team performing the epidural technique had no other involvement in the study. The clinical team caring for the patient (obstetricians, anesthesiologists, and nurses), outcome assessors, data analyst, and study participants were blinded to randomization. A blinded investigator (N.S. or M.W.) assessed all perioperative outcomes.

### Epidural Placement

As described in a prior study,^8^ the epidural procedure was performed before entering the operating room, to allow epidural analgesia to be established before conversion to cesarean delivery anesthesia with epidural chloroprocaine, as would be the case during an urgent cesarean delivery. Approximately 1 hour before scheduled cesarean delivery, participants received the epidural in their room on the labor and delivery ward according to the randomization assignment. Participants received a 500 mL co-load of lactated ringers solution and routine American Society of Anesthesiologists–recommended monitoring. The epidural was performed in the sitting position at the L3/4 or L4/5 interspace via the midline approach using a 17-gauge Touhy needle and a loss of resistance to saline technique. In the DPE group, the dura was punctured with a 25-gauge, pencil-point needle (Pencan; B Braun) using a needle-through-needle technique, and spontaneous return of cerebrospinal fluid was confirmed. In both groups, a flexible 19-gauge, spring closed-tip catheter (Perfix; B Braun) was inserted 5 cm into the epidural space. A test dose of 3 mL of 1.5% lidocaine with 1:200 000 epinephrine was injected to exclude intravascular or intrathecal placement. Approximately 5 minutes after a negative test dose, participants received up to 20 mL of 0.0625% bupivacaine with 2 μg/mL fentanyl (in 5-mL aliquots) to establish a bilateral sensory level to pinprick at the T10 dermatome. The sensory level was maintained with a continuous epidural infusion of the same solution (Cadd-Solis; Smiths Medical) at 12 mL per hour until time of entry into the operating room.

### Protocol for Epidural Extension Anesthesia

A blinded investigator (N.S. or M.W.) confirmed that participants had at least a bilateral T10 sensory level preoperatively. Alternative neuraxial anesthesia was offered if the block had not reached a T10 level. Participants were then transferred to the operating room, and routine monitoring was reapplied. The sensory and motor block were assessed according to the Modified Bromage score.^9^ The fetal heart rate was monitored intermittently until commencement of surgery.

Epidural extension anesthesia was standardized on the basis of institutional practice. A co-load of lactate ringers solution (500 mL) was given intravenously over approximately 15 minutes. After confirming negative aspiration of the epidural catheter, a test dose of 5 mL of 3% chloroprocaine was given, and the patient was monitored for signs of accidental intrathecal injection. If no abnormal signs were observed after 3 minutes, a further 15 mL of 3% chloroprocaine was injected over approximately 1 minute. The start of this injectate was defined as time zero and the beginning of epidural extension. A blinded investigator (N.S. or M.W.) assessed the block at 1-minute intervals, initially moving from a caudal to a cranial direction and then more frequently as the block ascended. A Neurotip (Owen Mumford) provided a standardized sharp-pain stimulus.

If a T6 bilateral block was not achieved within 10 minutes after the start of epidural extension, then 5-mL aliquots of epidural 3% chloroprocaine were administered every 5 minutes up to a total maximum cumulative dose of 30 mL. If the block did not reach a T6 level within 20 minutes, the attending anesthesiologist provided a different anesthetic technique according to clinical discretion. A phenylephrine infusion was started at 25 µg per minute, and the dose was titrated to maintain systolic blood pressure within 15% of preoperative baseline.

Intraoperatively all patients received intravenous cefazolin (2 g), dexamethasone (4 mg), ondansetron (4 mg), and ketorolac (30 mg) in the absence of contraindications. After delivery of the infant, an intravenous oxytocin infusion was commenced, and 3 mg of epidural morphine was administered for postoperative analgesia, followed by oral acetaminophen (650 mg every 6 hours), ibuprofen (600 mg every 6 hours), and oxycodone (5-10 mg every 4 hours) for breakthrough pain as required.

### Outcomes

The primary outcome was the onset time to surgical anesthesia. This was defined as the start of epidural extension anesthesia (time zero on the stopwatch) to when the patient could no longer feel sharp sensation at T6 (assessed bilaterally at the midclavicular line).

Secondary outcomes included the quality of epidural anesthesia. This was a composite of the following factors: (1) failure to achieve a T10 bilateral block preoperatively (after epidural administration of 3 mL of 1.5% lidocaine with 1:200 000 epinephrine and up to 20 mL of 0.0625% bupivacaine with 2 µg/mL fentanyl), (2) failure to achieve a surgical block within 15 minutes of epidural extension anesthesia, (3) the requirement for intraoperative analgesia supplementation, (4) requirement for repeat neuraxial procedure, or (5) conversion to general anesthesia. The intraoperative analgesia supplementation rate was defined as the requirement for any rescue medication to control discomfort or pain during cesarean delivery. The choice of medication was at the clinical discretion of the anesthesiologist. All components of the composite outcome were treated as binary measures, and the presence of any event was considered positive for the secondary outcome (collapsed composite).

Other secondary outcomes included maternal satisfaction and pain score during surgery (assessed in recovery room, where 0 indicates very dissatisfied and 10 indicates very satisfied), opioid consumption in the first 24 hours, adverse events (eg, self-reporting of nausea, vomiting, or pruritus), timings (from primary outcome to start of surgery and duration of surgery), maternal vasopressor requirements (intraoperative dose administered), epidural block assessments, neonatal outcomes (Apgar score at 1 and 5 minutes and the umbilical cord blood gasses), and other adverse events, including local anesthetic systemic toxicity, accidental dural puncture (ADP), high spinal (ie, exaggerated cranial spread of spinal anesthesia leading to hand weakness, difficulties breathing, and, finally, unconsciousness), and postdural puncture headache (PDPH) assessed on postpartum day 1.

### Statistical Analysis

The primary outcome of the study was time to loss of sharp sensation at the T6 level. On the basis of our previous study,^8^ we anticipated that participants in the standard epidural group would have a mean onset to surgical anesthesia of approximately 10 minutes. We estimated that a difference in onset times of at least 2 minutes between the standard epidural and DPE would represent a clinically important difference. Thus, a total of 120 mother-infant dyads would provide 90% power of detecting a difference with an α level of .05, assuming a common SD of 3 minutes. Therefore, we would need 60 mother-infant dyads in each group of the trial. In total, we planned to enroll 140 mother-infant dyads to account for withdrawals and protocol violations. The primary analyses were based on an intention-to-treat analysis. All tests were 2-tailed and used a significance level of *P* < .05. Data are reported as median (IQR) or number (percentage) as appropriate. Onset time to loss of sharp sensation at the T6 dermatome were compared between groups using Kaplan-Meier estimates and log-rank tests. Participants who never achieved a sensory block at T6 level were administratively censored at 20 minutes after the start of epidural extension. Cox proportional hazard models were used to compare the rate of loss of sharp sensation at the T6 level between groups adjusting for age, body mass index (calculated as weight in kilograms divided by height in meters squared), race and ethnicity, indication for surgery, epidural loading dose in labor and delivery room, duration of epidural infusion, and preoperative sensory and motor block. The secondary composite outcome of quality of epidural anesthesia was analyzed using univariate logistic regression. Wilcoxon rank-sum tests were used to compare the groups with respect to continuous data. χ^2^ or Fisher exact tests were used to compare the groups with respect to binary or categorical outcomes where appropriate. All analyses were performed using STATA statistical software version 14.0 (StataCorp).

## Results

**Figure 1.**
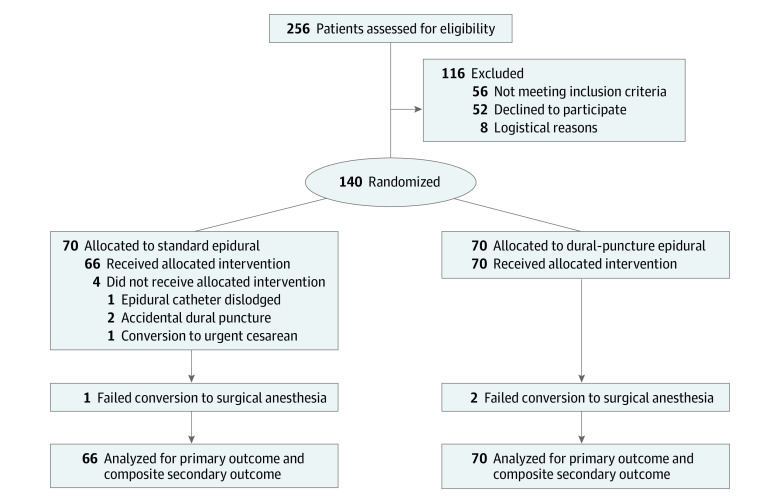
Study Flow Diagram

Among 256 parturients who were assessed for eligibility, 140 participants (mean [SD] age, 30.1 [5.2] years) were enrolled into the study (70 participants were randomized to each group) (Figure 1). Four patients in the standard epidural group were excluded because of epidural catheter dislodgement (1 patient), ADP (2 patients), and conversion to urgent cesarean delivery (1 patient). Therefore, 66 patients in the standard epidural group and 70 patients in the DPE group were included in the primary outcome assessment. One woman in the standard epidural group and 2 women in the DPE group failed to achieve a T6 sensory level (ie, failed epidural extension anesthesia) after receiving the maximum dose of 30 mL of 3% chloroprocaine. Alternative neuraxial anesthesia was successful for all patients, and their data are included as part of the intention-to-treat analysis. Table 1 presents baseline participant characteristics, indications for cesarean delivery, and preoperative epidural variables.

**Table 1.** Demographic and Clinical Characteristics of Participants

Characteristic	Patients, No. (%)	
	Standard epidural (n = 66)	Dural-puncture epidural (n = 70)
Age, mean (SD), y	31.0 (5.1)	29.1 (5.3)
Height, mean (SD), kg	163.7 (7.4)	162.0 (6.5)
Weight, median (IQR), kg	85.5 (78-99.3)	86.9 (74.8-101.2)
Body mass index, mean (SD)^a^	32.9 (4.9)	33.9 (6.4)
Race		
Black	19 (28.8)	27 (38.6)
White	45 (68.2)	40 (57.1)
Other^b^	2 (3.0)	3 (4.3)
Gestational age, median (IQR), d	273 (268-274)	273 (268-274)
Surgical indication		
Repeated LTCD	57 (86.4)	48 (68.6)
Breech	5 (7.6)	4 (5.7)
Maternal request	0	1 (1.4)
Previous shoulder dystocia	0	1 (1.4)
HIV positive	0	1 (1.4)
Cephalopelvic disproportion	1 (1.5)	2 (2.9)
Placenta previa	2 (3.0)	2 (2.9)
Previous myomectomy	0	2 (2.9)
Herpes simplex	0	1 (1.4)
Fetal indications	1 (1.5)	8 (11.4)
Surgical procedure		
Primary LTCD	9 (13.6)	18 (25.7)
Repeated LTCD	40 (60.6)	33 (47.1)
Repeated LTCD and bilateral tubal ligation	17 (25.8)	19 (27.1)
Epidural loading dose		
10 mL	5 (7.7)	13 (13.9)
15 mL	18 (27.7)	20 (28.6)
20 mL	42 (64.6)	37 (52.9)
Epidural infusion duration, median (IQR), min	36 (30-68)	45 (30-80)

Abbreviation: LTCD, low transverse cesarean delivery.

^a^ Body mass index is calculated as weight in kilograms divided by height in meters squared.

^b^ Includes Asian, Hispanic ethnicity, and any other race or ethnicity not otherwise specified.

### Primary Outcome: Onset Time to Surgical Anesthesia

**Figure 2.**
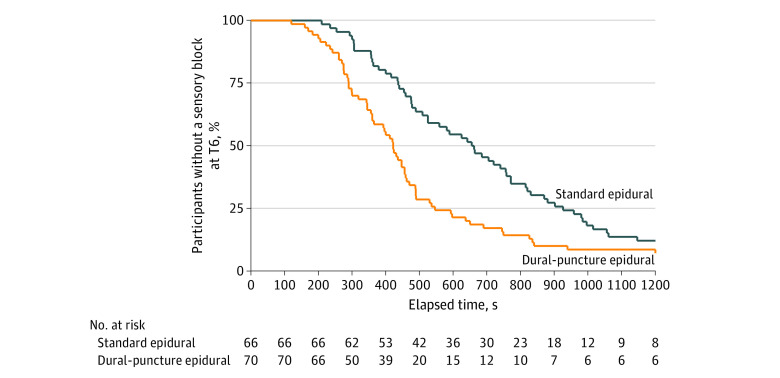
Kaplan-Meier Survival Curves for the Primary Outcome Kaplan-Meier survival curves for the primary outcome show proportion of patients who did not achieve a sensory blockade level to T6.

The primary outcome of median (IQR) time to surgical anesthesia was 655 (437-926) seconds in the standard epidural group and 422 (290-546) seconds in the DPE group (Table 2). The median (IQR) difference in the onset time of sensory block between the 2 groups was 233 (104-369) seconds. There was a significant difference in the time required to achieve surgical anesthesia between DPE and standard epidural (hazard ratio, 1.84; 95% CI, 1.29-2.64; *P* < .001). In Figure 2, a Kaplan-Meier survival curve demonstrates the onset time to a T6 sensory block. A post hoc sensitivity analysis adjusting for maternal characteristics (age, BMI, race, and indication for cesarean delivery) and epidural block characteristics (epidural loading dose used to initiate analgesia, duration of preoperative epidural infusion, sensory level, and motor block at the time of epidural extension anesthesia) did not significantly alter the results (hazard ratio, 1.74; 95% CI, 1.18-2.57; *P* = .005).

**Table 2.** Intraoperative Data, Outcomes, and Adverse Events

Outcome	Patients, No. (%)		Difference	*P* value
	Standard epidural (n = 66)	Dural-puncture epidural (n = 70)		
Onset time to a T6 sensory block, primary outcome, median (IQR), s	655 (437 to 926)	422 (290 to 546)	−233 (−369 to −104)	<.001
Sensory block on entry into the operating room, vertebral range				
T7-T9	23 (38.3)	37 (54.4)	[Reference]	.07
T10-T12	37 (61.7)	31 (45.6)	0.5 (0.3 to 1.1)^a^	
Modified Bromage Score on entry into the operating room^b^				
0	46 (71.9)	48 (69.6)	[Reference]	.77
1-2	18 (28.1)	21 (30.4)	1.1 (0.5 to 2.4)^a^	
Modified Bromage Score when primary end point achieved^b^				
1	1 (1.7)	6 (9.0)	[Reference]	.12
2-3	57 (98.3)	61 (91.0)	0.2 (0.02 to 1.5)^a^	
Epidural extension to start of surgery, median (IQR), min	20 (17 to 23)	20 (15 to 22)	0 (−2 to 2)^c^	.30
Surgery duration, median (IQR), min	37 (33 to 44)	40 (32 to 56)	4 (−2 to 9)^c^	.27
Phenylephrine, median (IQR), µg	22685 (1400 to 3210)	2265 (1630 to 316)	−3 (−602 to 597)^c^	.69
Ephedrine use				
0	48 (82.8)	50 (76.9)	[Reference]	.63
5	6 (10.3)	6 (9.2)	1.0 (0.3 to 3.2)^a^	
10	2 (3.5)	5 (7.7)	2.4 (0.4 to 13.0)^a^	
20	0	1 (1.5)	NA	
25	1 (1.7)	3 (4.6)	2.9 (0.3 to 28.7)^a^	
35	1 (1.7)	0	NA	
Intraoperative pain^d^				
No pain	53 (81.5)	64 (92.6)	[Reference]	.17
Mild (1-3)	1 (1.5)	1 (1.5)	0.8 (0.1 to 13.6)^a^	
Moderate (4-7)	5 (7.7)	1 (1.5)	0.2 (0.02 to 1.5)^a^	
Severe (8-10)	6 (9.2)	3 (4.4)	0.4 (0.1 to 1.7)^a^	
Time from induction of anesthesia to patient discomfort, min	32 (29 to 41)	28 (25 to 41)	−4 (−29 to 21)^c^	.53
Intraoperative analgesia				
No intraoperative analgesia	54 (81.8)	63 (90)	[Reference]	.63
Opioid (IV)	2 (3.0)	1 (1.4)	0.4 (0.04 to 4.9)^a^	
Epidural local anesthesia	4 (6.1)	1 (1.4)	0.2 (0.02 to 2.0)^a^	
Ketamine (IV)	1 (1.5)	1 (1.4)	0.9 (0.1 to 14.0)^a^	
Combination therapy	5 (7.6)	4 (5.7)	0.7 (0.2 to 2.7)^a^	
Adverse events				
Nausea	17 (29.8)	21 (31.8)	1.1 (0.5 to 2.4)^a^	.81
Vomiting	7 (12.1)	3 (4.6)	0.4 (0.1 to 1.4)^a^	.19
Pruritus	7 (12.1)	6 (9.1)	0.7 (0.2 to 2.3)^a^	.59
Shivering	12 (20.7)	10 (15.2)	0.7 (0.3 to 1.7)^a^	.42
Oxycodone use in the first 24 h after surgery	55 (83.3)	63 (90)	1.8 (0.7 to 5.0)^a^	.25
Cumulative oxycodone, mean (SD), mg	24.3 (15.8)	20.1 (15.3)	−4.2 (−9.9 to 1.5)^e^	.14
Patient satisfaction^f^				
0-3	1 (1.7)	1 (1.5)	NA	.85
4-7	4 (6.9)	3 (4.6)	NA	
8-10	53 (91.4)	61 (93.9)	NA	
Apgar score at 1 min				
<8	6 (10.5)	13 (19.1)	[Reference]	.18
9-10	51 (89.5)	55 (80.9)	0.5 (0.2 to 1.4)^a^	
Apgar score at 5 min				
<8	1 (1.8)	5 (7.4)	[Reference]	.22
9-10	55 (98.2)	63 (92.7)	0.2 (0.03 to 2.02)^a^	
Umbilical vein pH, mean (SD)	7.3 (0.05)	7.3 (0.06)	−0.01 (−0.03 to 0.01)^e^	.55
Umbilical artery pH, mean (SD)	7.2 (0.06)	7.2 (0.07)	−0.01 (−0.04 to 0.01)^e^	.47
Umbilical vein BE, mean (SD)	−3.0 (1.7)	−4.3 (2.8)	−1.2 (−2.1 to −0.4)^e^	.006
Umbilical artery BE, mean (SD)	−3.1 (2.3)	−4.4 (2.8)	−1.4 (−2.3 to −0.4)^e^	.007
Other adverse events				
High spinal	1 (1.5)	3 (4.3)	2.9 (0.2 to 155.1)^a^	.34
Local anesthesia systemic toxicity	0	0	NA	

Abbreviations: BE, base excess; IV, intravenous; NA, not applicable.

^a^ Data are odds ratio (95% CI).

^b^ The Bromage score is as follows: 0, no motor block, full flexion of knees and feet; 1, just able to move knees; 2, able to move feet only; and 3, unable to move feet or knees.

^c^ Median difference is shown.

^d^ The intraoperative pain scale ranged from 0 (no pain) to 10 (worst pain).

^e^ Mean difference is shown.

^f^ The patient satisfaction rating ranged from 0 (extremely dissatisfied) to 10 (extremely satisfied).

### Secondary Outcomes: Quality of Epidural Anesthesia

**Figure 3.**
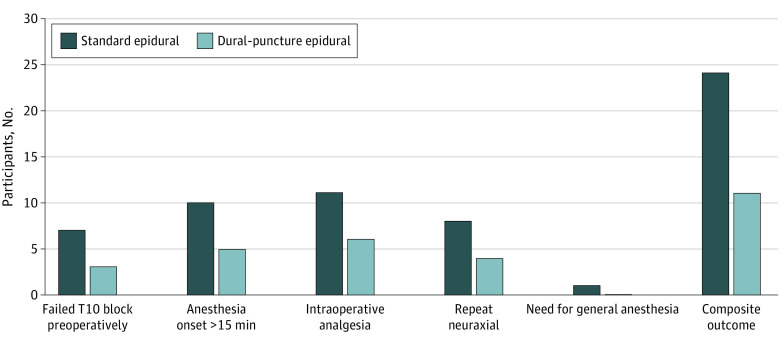
Secondary Composite Outcome for Quality of Anesthesia and Its Individual Components

For the composite of 5 outcomes indicating lower quality of anesthesia, the observed composite rate was 11 of 70 women (15.7%) in the DPE group compared with 24 of 66 women (36.3%) in the standard epidural group (odds ratio, 0.33; 95% CI, 0.14-0.74; *P* = .007). DPE was associated with a more favorable quality of epidural blockade (18.6% risk reduction) for cesarean delivery. Figure 3 provides a summary of quality of anesthesia outcomes and includes a breakdown of each individual component.

Table 2 summarizes secondary outcomes including maternal and neonatal outcomes. No differences were demonstrable between groups except for an increased base deficit in the DPE group. There were no reports of local anesthetic systemic toxicity or neurological complications. Among the 2 patients who had an ADP, 1 woman developed a PDPH and received an epidural blood patch with resolution of her symptoms. The other woman had an intrathecal catheter placed at the time of ADP, which was used for cesarean delivery anesthesia. She did not require a blood patch and was discharged on postoperative day 2. One patient in the standard epidural group and 3 patients in the DPE group had a high spinal that did not require intervention (Table 2).

## Discussion

The main finding from this RCT is that epidural extension initiated with a DPE has an approximately 3-minute faster onset time to cesarean anesthesia than a standard epidural and a more favorable quality of epidural blockade (18.6% risk reduction) for cesarean delivery. We believe these results are clinically meaningful and relevant for patients requiring emergent cesarean delivery under epidural extension anesthesia. A recent study by Palmer et al^10^ reported operating room to incision times for category 1 cesarean deliveries (ie, immediate threat to life of the woman or fetus) based on the anesthetic technique performed. General anesthesia was found to have the most rapid times from operating room to incision (6 minutes) but was also associated with the worst neonatal outcomes, whereas onset times in the epidural top-up group were longer (11 minutes).^10^ Our results indicate that initiating labor analgesia using a DPE technique may achieve onset times similar to those for general anesthesia and may, therefore, reduce the need for general anesthesia conversion in an emergency. To evaluate the time required to extend labor epidural analgesia to anesthesia for an intrapartum cesarean delivery, we first provided epidural analgesia using a regimen similar to that provided to laboring patients, and then evaluated the onset of anesthesia for cesarean delivery. Although this is not how anesthesia is performed in the clinical setting of scheduled cesarean delivery cases, this experiment provides important information. The use of a test dose followed by bolus administration of epidural chloroprocaine was chosen because it closely represents the clinical environment of rapid conversion of labor epidural analgesia to surgical anesthesia for emergent cesarean delivery. A recent RCT^11^ compared the efficacy of de novo DPE and standard epidural for repeated scheduled cesarean and reported a 1.9-minute difference to T6 sensory level between the groups in favor of the DPE cohort. That trial also found that women in the DPE group required significantly less intraoperative analgesia supplementation (28.1% vs 12.1%).^11^ These results are consistent with our findings demonstrating faster onset time and improved block quality with the DPE technique.

Although our study demonstrates clear evidence of benefit in women requiring elective cesarean delivery, studies examining labor analgesia have reported inconsistent findings. Studies^4,7,12,13,14^ have reported a lower incidence of unilateral and inadequate labor analgesia, reduced need for manual top-ups, modest improvement in onset of analgesia, and improved sacral coverage. A recent meta-analysis^15^ from 2022 reported faster onset to satisfactory pain relief in women receiving DPE labor analgesia. Since then, a large RCT compared the standard epidural technique with the DPE technique in parturients with obesity.^5^ This patient population could potentially benefit the most from DPE-initiated analgesia; however, the investigators found no significant difference in quality of analgesia between the 2 techniques.^5^

These contradictory results may be due to methodological heterogeneity. The mechanisms determining flux of local anesthetics through the meninges and the hole created by the dural puncture are complex and have not yet been fully elucidated.^16,17,18,19^ The size of the dural puncture is important, but there are likely other important factors involved, such as the total drug mass of the injectate, timing since DPE was performed, epidural bolus administration, and the injectate pressure generated.

The physicochemical properties of the local anesthetic may also be an important determinant of transmeningeal diffusion. Chloroprocaine is mostly ionized at physiological pH with an inherently slow diffusion rate. Our study indicates that the flow of chloroprocaine may have been promoted through the dural puncture owing to the rapid administration of a relatively large mass of drug.

The differences in umbilical cord base excess between the groups warrant further study, as do the differences in Apgar scores, although they were not significant. This study was underpowered for these outcomes, and the clinical relevance of such findings is questionable because umbilical artery and vein pH values were within normal limits.

### Limitations

We acknowledge that this study has some limitations. First, and most importantly, the epidural technique was performed in women who were scheduled for elective cesarean delivery and were not in labor. The epidural technique was performed approximately 1 hour before entry into the operating room, and an epidural infusion was used to maintain a T10 sensory level.^8^ Little is known about the patency of the dural hole created following a DPE, which may diminish over time and limit the translocation of drugs from the epidural to intrathecal space. Future studies should compare the DPE and standard epidural techniques for intrapartum cesarean delivery to determine whether the benefits of DPE-initiated analgesia persist. Second, recent guidelines recommend light touch as the primary testing modality to a level of T5 or higher to reduce the risk of intraoperative pain, and, therefore, this may also influence the onset time to surgical anesthesia.^20^ Third, our study was not powered to detect complications occurring less frequently, such as PDPH and ADP. This is important because many anesthesiologists will use a 27-gauge needle as part of a combined spinal-epidural technique to reduce the risk of PDPH. Fourth, our results may differ if other local anesthetics and opioids combinations, such as lidocaine or ropivacaine with fentanyl, are used. Chloroprocaine was chosen in this study because of its ease of administration without the need for opioids and other additives along with the low risk of systemic toxic effects, which favors rapid administration for emergent cesarean delivery.

## Conclusions

In the context of our study, a DPE performed within an hour of epidural extension for elective cesarean delivery was associated with a faster onset of anesthesia, improved block quality, and a favorable risk-to-benefit ratio compared with standard epidural. Further studies are needed to investigate whether these findings can be validated in the clinical context of intrapartum cesarean deliveries, whether these benefits persist with different local anesthetic mixtures and timings from DPE to epidural extension, and to confirm neonatal outcomes.

## Notes

Supplement 1.
Trial Protocol

Supplement 2.
Data Sharing Statement
