# Supplementary material for: Effect of Dural-Puncture Epidural vs Standard Epidural for Epidural Extension on Onset Time of Surgical Anesthesia in Elective Cesarean Delivery: A Randomized Clinical Trial
Source: JAMA Netw Open. 2023 Aug 1;6(8):e2326710. doi: 10.1001/jamanetworkopen.2023.26710 (PMC10394571; doi:10.1001/jamanetworkopen.2023.26710)

Study Title: Dural Puncture Epidural versus Epidural anesthesia for cesarean delivery: A randomized, double-blind study

Principal Investigator: Nadir Sharawi, MD
University of Arkansas for Medical Sciences
4301 W. Markham Street, Slot # 515
Little Rock, AR 72205
Telephone: (501) 319-2622
Email: NElsharawi@uams.edu

Sub-Investigator (s):

Jill Mhyre, MD
University of Arkansas for Medical Sciences
4301 W. Markham Street, Slot # 515
Little Rock, AR 72205
Telephone: (501) 554-4943
Email: JMMhyre@uams.edu

Matthew Williams, MD
University of Arkansas for Medical Sciences
4301 W. Markham Street, Slot # 515
Little Rock, AR 72205
Email: MWilliams@uams.edu

Study location: University of Arkansas for Medical Sciences
4301 W. Markham Street, Slot # 515
Little Rock, AR 72205

Table of Contents

Table of Contents 2
Abbreviations 4
Background and Rationale 5
Specific Aims 5
Primary Outcome 6
Secondary Outcomes 6
Exploratory Outcome 6
Screening/Baseline Phase 7
Pre-operative Phase 8
Intra-operative Phase 8
Intra-operative Monitoring 10
Concurrent Medication 10
Assessment of Primary Efficacy Parameters 11
Epidural Study Solutions 11
Post-operative Phase 11
Inclusion Criteria 11
Exclusion Criteria 12
Accrual Goal 12
Recruitment Plan 12
Risks and Benefits 12
Benefits of DPE/ Epidural anesthesia 12
Risks of Epidural/DPE anesthesia 13
Study Medication Risks 13
Risk Mitigation 14
Drug Accountability and Subject Compliance 14
Data Handling and Recordkeeping 14

Data Analysis 14
Sample Size 15
Randomization 15
Withdrawal of Participants 15
Stopping the Study 15
Ethical Considerations 16
Dissemination of Data 16
Appendices 18

Abbreviations

ASA	American Society of Anesthesiologist
BP	Blood Pressure
CD	Cesarean delivery
CSE	Combined spinal epidural
CSF	Cerebro-Spinal Fluid
DPE	Dural Puncture Epidural
ECG	Electrocardiogram
HIPAA	Health Insurance Portability and Accountability Act
IRB	Institutional Review Board
LA	Local anesthetic
L&D	Labor and Delivery
Mcg	Microgram
Min	Minute
ml, mls	Milliliter, Milliliters
PACU	Post Anesthesia Care Unit
SOC	Standard of care
T5	Thoracic Dermatome Level 5
T6	Thoracic Dermatome Level 6
UAMS	University of Arkansas for Medical Sciences
VAS	Visual Analogue Scale

Background and Rationale

Cesarean delivery (CD) is the most commonly performed surgical procedure worldwide. Indications for CD include maternal or fetal distress, cervical arrest of dilation and elective CD. The rate of CD rose from 20.7% in 1996 to 32.9% in 2009 in US [1]. The rate of CD nationally currently is 32% of all births [2]. CD can be performed under neuraxial (epidural, spinal or combined spinal-epidural {CSE}) or general anesthesia. Based on expert consensus and clinical evidence, neuraxial anesthesia has been recommended over general anesthesia for by the American Society of Anesthesiologists and the Society for Obstetric Anesthesia and Perinatology [3].

Spinal anesthesia is limited by complications like toxicity of local anesthetic agents, transient neurologic back pain, post-dural puncture headache, nerve injury, caudal equina syndrome and spinal hematoma [4]. While epidural anesthesia is limited by slow onset of sensory block and difficulty with achieving bilateral analgesia that may require repeated adjustment of the epidural catheter [5]. Dural puncture epidural (DPE) is a newer technique increasingly used for labor analgesia to overcome these limitations. It involves the creation of a single dural perforation with a spinal needle, introduced through an epidural needle (similar to a CSE), but without the administration of medications through the spinal needle. This technique was developed to address the limitations of both epidural and spinal anesthesia when performed for the purpose of providing pain relief to laboring women.

When compared to an epidural technique, DPE has been shown to decrease manipulation of the epidural catheter, provide a better and earlier onset of labor analgesia [6,7,8] , a lower incidence of failure [9], improved bilateral block and a lower incidence of intra-op local anesthetic bolus requirement [10]. To date most of the studies have utilized DPE for the purposes of labor analgesia. Only one study has evaluated the use of DPE for surgical anesthesia for lower abdominal surgery [11]. The aim of this randomized double-blind study is to compare DPE with epidural anesthesia in the setting of elective CD.

Specific Aims

The aim of this study is to compare the onset time of anesthesia between standard epidural and DPE in elective cesarean delivery. We hypothesize that a DPE technique with a 25-gauge spinal needle will have a faster onset and improved quality of surgical anesthesia when compared to a standard epidural.

Primary Outcome

The primary outcome will be the onset time of surgical anesthesia. This will be measured from the induction of anesthesia (as defined by the beginning of injection of the “Induction 1 syringe”) to the point at which sharp sensation is lost bilaterally at the T6 dermatomal level (as measured by a blunt plastic neurotip® (Owen Mumford, USA) device).

Secondary Outcomes

1) "Inadequate Neuraxial Anesthesia":
This composite outcome (any or none) will defined as the failure to achieve at least a T10 bilateral sensory level pre-operatively (after 3 ml 1.5% lidocaine with 1:200,000 epinephrine 45 mg lidocaine and up to 20 ml of 0.0625% bupivacaine), the requirement for intraoperative analgesia supplementation, conversion to general anesthesia or repeat neuraxial procedure, or failure to achieve the primary outcome within 15 minutes between the two groups.

2) We will compare the intraoperative supplementation rate between the two groups. This is defined as the percentage of women who require any additional medications to control pain during the elective CD in each arm of the trial.

Exploratory Outcome

The following will be abstracted from the medical records or reported by the patient in the perioperative period:

- Maximum pain Visual Analogue Scale (VAS) during surgery (as reported by patient, scored from 0-10 in the PACU).
- Incidence of side effects:
  - Nausea (self-reported by patient, yes or no).
  - Vomiting (observed yes or no).
  - Itching (self-reported by patient, yes or no).
- Use and dose of vasopressors (phenylephrine and ephedrine)
- Overall patient satisfaction score (asked and scored from 0-10).
- Neonatal Apgar scores (from medical records).
- Umbilical cord blood gases taken after delivery (arterial and or venous – from medical records).
- Opioid consumption over 24 hours postoperatively (from medical records)

Flow Chart

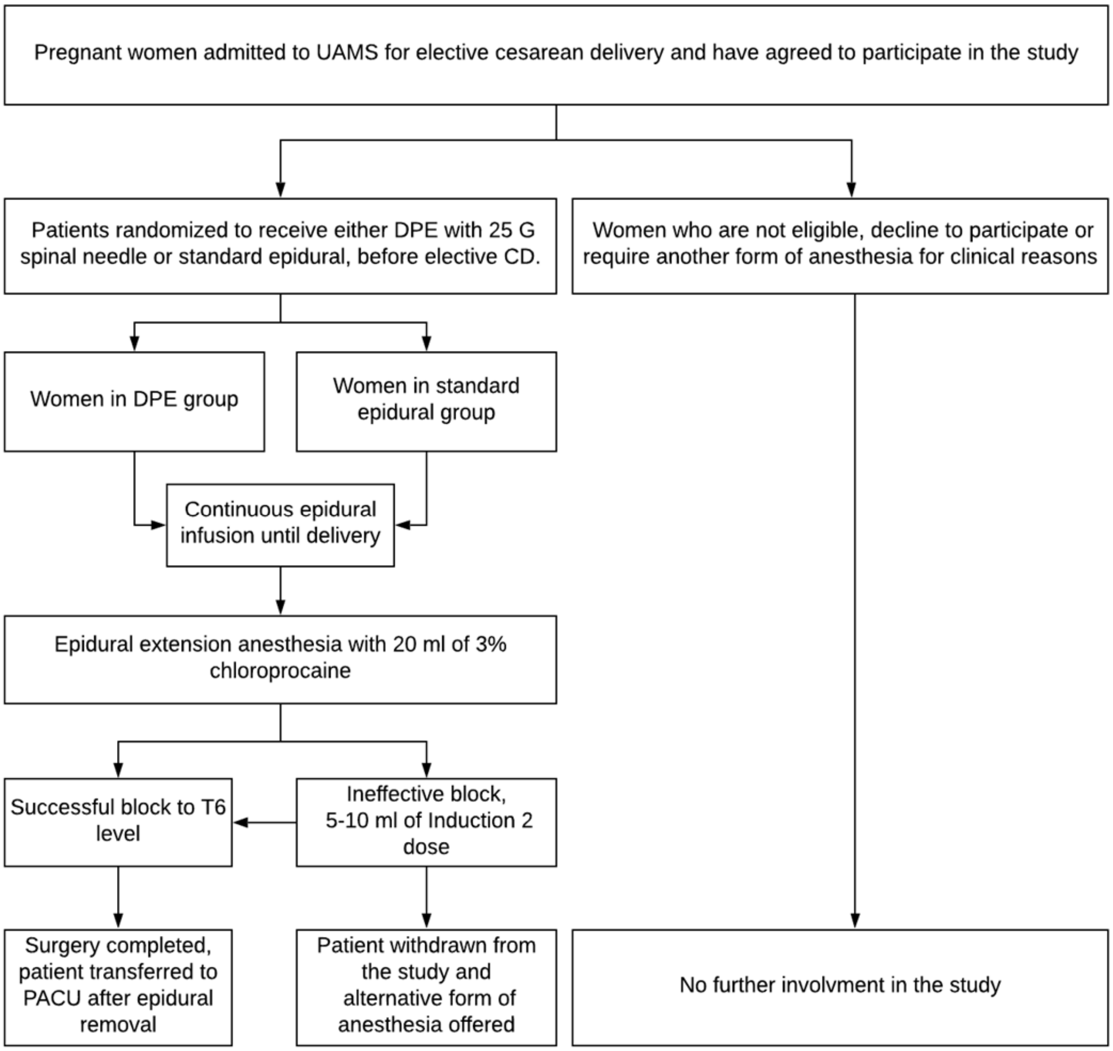

Study Design and Procedure

<u>Screening/Baseline Phase</u>

The anesthesiologists performing the pre-operative evaluation (standard of care; SOC) will alert a member of the study team if the patient meets the inclusion criteria for the study. Following informed consent, we will obtain demographic and clinical information including, but not limited to, height, weight, age, current medications, medical diagnoses, and history of anesthesia complications (all SOC). Standard non-invasive

vital signs (heart rate, blood pressure, respiratory rate, and temperature) will be obtained from the pre-operative work up.

<u>Pre-operative Phase</u>

Patients who have been enrolled into the study will be randomized to receive either DPE or standard epidural. As is standard practice, the informed consent process will be undertaken before any mood alerting medications are administered. This procedure will be performed in the patient's room upon admission to the Labor & Delivery Suite (L&D) shortly before (usually 1 hour) their scheduled time for cesarean section. Epidural/ DPE will be performed by an un-blinded anesthesiologist. The un-blinded anesthesiologist will have no other role in the patient's care other than performing the procedure. After insertion of the epidural or DPE, a low dose local anesthetic infusion will be infused into the epidural catheter up until the time of surgery (bupivacaine 0.0625% with 2 mcg/ml fentanyl; SOC). We have previously performed a similar study (IRB # 207313) with great success. Participant would then move on to the next phase of the study (see below). Patients who are not enrolled in the study would normally receive either an epidural or DPE in the same manner. The choice of anesthetic technique for the non-study patients is dependent on the preference of the anesthesiologist and clinical context.

<u>Epidural or DPE study group</u>

Participants will be blinded to which group they are being assigned. Participants are unable to see the procedure (due to placement in the lower back). Both procedures are almost identical except for a minor variation in technique. As such the time taken and "feel" of the procedure are identical. The unblinded anesthesiologist will insert the epidural or DPE based on randomization. They will have no further role in the study after the procedure. Insertion of the epidural or DPE will follow the standard practices in which all epidurals/DPE are inserted.

<u>Intra-operative Phase</u>

The patient will be transferred from her room to the operating room at the time of scheduled surgery. The epidural pump will be discontinued, and anesthesia care will be conducted in the same manner as all cesarean deliveries under epidural extension anesthesia (this refers to the process of providing anesthesia using a pre-existing epidural/DPE). Anesthesia will be induced in the standard manner. Motor and sensory block will be tested at the end of the epidural loading dose. Loss of sharp sensation will be measured using a blunt plastic neurotip® (Owen Mumford, USA) until the sensation of "sharpness" at the T5 dermatomal level has been reached. The neurotip is a noninvasive medical device that we use routinely to assess the level of anesthesia for

CD. The T6 level measured at the xiphoid process (which is an easily palatable bony landmark) will be marked bilaterally with a washable marker pen to guide assessment of the primary end point. Sensory testing will be performed from caudad to cephald (i.e. from blocked to unblocked dermatomes) to identify the first unblocked dermatome.
To identify the level where the sensation of sharp is first appreciated, the investigator will ask the question: "Tell me when you feel the sensation of something sharp touching your skin." Both the motor and sensory block evaluations are part of the standard clinical care of patients receiving neuraxial anesthesia. The main difference for participants enrolled in the study is that the frequency of sensory assessments will be increased so that the onset of surgical anesthesia can be accurately documented (approximately every minute and then more frequently as the sensory block approaches the primary end point).

The local anesthetic solution will be given in three phases (SOC for epidural/DPE extension anesthesia):

1. Test dose – to check for correct placement of epidural
2. Induction dose 1 – to induce anesthesia
3. Induction dose 2 – further dose of local anesthesia if required (as per instructions below)

A second anesthesiologist, blind to the type of block will manage the clinical care of the patient from the beginning of the study (after epidural catheter placement) and will administer the induction drug (prepared by that anesthesiologist as per SOC). There will be no difference in this clinician's care of the subject than if she were not enrolled in the study. They will assess the onset of anesthesia and manage all aspects of the subject's clinical care including the documentation of the local anesthetic (LA) solution administration timing and its clinical effects. The speed of onset will be assessed from the end of epidural test dose. This will be defined as time zero and the start of anesthesia. The primary outcome will be the time taken to lose sharp sensation from a neurotip/pen device at the thoracic dermatome level 6 (T6). See below for a description of this assessment.
The primary outcome will be documented on a separate data collection tool (which the un-blinded anesthesiologist will not have access to). If required, intra-operative analgesia will be offered in the form of further epidural top-up, intravenous fentanyl, ketamine, nitrous oxide or replacement of neuraxial anesthesia/conversion into general anesthesia at the Standard of care (SOC). These are all commonly used medications that provide pain relief during cesarean sections in the event of breakthrough pain. The choice of which drug to use is at the discretion of the anesthesiologist. This information will be abstracted from medical records. In the event of an emergency situation blinding

would be broken and the treating anesthesiologist would be informed to what group the patient belongs.

<u>Local anesthetic solution for anesthesia</u>

Below is the description of the SOC preparation & administration of local anesthetic and conduct within the operating room for epidural extension anesthesia in parturients that require CD. 20 ml of 3% chloroprocaine which will be drawn up into a 20 ml syringe At the start of epidural extension anesthesia, a 5 ml test dose will be administered through the epidural (as previously described above). After three minutes, if there are no signs of accidental spinal block or intravascular administration then the remaining 15 ml of the Local Anesthetic solution will be administered.

A further 5 ml of chloroprocaine will be administered if Induction dose 1 is not effective in providing sufficient anesthesia (reaching primary end point) after 10 minutes. This is referred to as Induction dose 2. If the primary end point is not reached at the 15-minute mark (total elapsed time after giving Induction dose 1) then a final 5 ml of chloroprocaine can be given. If the primary end point is not reached within 20 minutes the subject will be withdrawn from the study and the anesthesiologist can induce anesthesia in whichever way they think is best. At this point the anesthesiologist will break blinding. Therefore, the total volume of local anesthesia that can be given to the patient at this stage is 30 ml (20 ml from Induction dose 1 and up to 10 ml from Induction dose 2 if necessary).

This above is our usual practice except for the following:

- We are being very precise in regard to documentation of timing (primary end point of study)
- Monitoring the sensory block more frequently
- Blinding and randomization as part of a clinical trial

Intra-operative Monitoring

As with all cesarean sections, full monitoring in the operating room will be applied and will be the same whether the participant is in the study or not.

Concurrent Medication

Subjects enrolled in the study will be treated as per normal practice for elective cesarean section. If a general anesthetic must be instituted, the subject’s participation in the study will be stopped.

Assessment of Primary Efficacy Parameters

Assessment will be made of the sensory level after the epidural induction dose. This will give an indication of the suitability of surgical anesthesia before proceeding with cesarean section. This will be assessed by the blinded clinical anesthesiologist with a Neurotip™ device, 2 minutes after the completion of the epidural top-up and then continuously if possible or at intervals of approximately 1 minute until a T6 level to sharp sensation is achieved. Motor function will be assessed using the Modified Bromage Score.

Epidural Study Solutions

The solutions and their administration procedures are identical to those used outside this research and are almost exclusively used for epidural extension anesthesia for non-scheduled cesarean delivery. All patients enrolled will receive the same study solution exactly prescribed as above which is our routine practice.

Post-operative Phase

Participants will be admitted to the PACU after completion of the operation. Care will be as per the SOC for all elective cesarean deliveries. Pain scores and cumulative opioid usage over the first 24 hours postoperatively, will be abstracted from the medical records of these subjects after discharge.
Before discharge from the PACU, the un-blinded anesthesiologist would access the patient's medical record and replace the charted "study group" with the either standard Epidural or DPE administered before closing the anesthetic record.

Study Population

All subjects scheduled for elective cesarean delivery will be screened for recruitment when admitted to UAMS labor and delivery unit. A member of the research team will approach the subject after completion of the anesthetic pre-assessment which is a standard of care.

Inclusion Criteria

Any patient requiring an elective cesarean section at UAMS labor and delivery unit who is:

- ≥ 18 years of age for the mother
- Singleton pregnancy
- Gestation > 36 weeks
- ASA class II and III
- Provides written consent

- Infant of mother
- Elective or non-urgent cesarean delivery

Exclusion Criteria

- Patient refusal
- Urgent/emergent cesarean sections
- ASA class IV or above
- Unable to understand English
- Significant back surgery or scoliosis
- Lethal fetal abnormality or likely to affect APGAR scores
- Weight > 120 kg
- Height < 150 cm
- Allergy to study solutions

Accrual Goal

A total of 140 mother-infant dyads (a total of 280 subjects) requiring an elective cesarean section at UAMS labor and delivery unit will be enrolled into the study.

Recruitment Plan

Potential subjects will be offered participation in the study after admission into the Labor and Delivery unit. All potential subjects will be informed of the study by a member of the study team after the anesthetic pre-operative consultation. The informed consent /HIPAA discussion will take place prior to any pre-operative medications being administered and the potential subject will be allowed as much time as necessary to consider participating in the study.

Risks and Benefits

The benefits and risks to the study participants overall will be the same as all patients presenting to L&D for elective cesarean delivery. That is, whether a patient decides to participate or not in the study, the normal standard of care is neuraxial anesthesia for CD as opposed to general anesthesia. The spinal, epidural and DPE are all commonly used in our unit to provide anesthesia for CD. The choice of anesthetic technique depends on the anesthesiologist's discretion.

Benefits of DPE/ Epidural anesthesia

These techniques will be considered together as they are similar. The main advantage of epidural/DPE anesthesia is the ability to extend anesthesia for as long as required. This also allows the administration of further local anesthetic solution through the

epidural if surgery is prolonged or to treat any episodes of intraoperative pain without having to convert to general anesthesia.

Risks of Epidural/DPE anesthesia

Risks to participants in this study due to epidural/DPE anesthesia is: Inadvertent intravascular injection or high epidural. This risk can be minimized by aspirating blood through the epidural catheter and administering a 'test dose' to rule out intravascular injection or accidental spinal administration that may lead to a high block. The above interventions are usually enough to minimize the risk of any hazards of intravascular injection or inadvertent spinal that may lead to a high block.

Disadvantages of participating in the study

Research related risk to study participants include the potential for loss of confidentiality. Measures to protect the confidentiality of study participants will be implemented as described in the Data Handling and Recordkeeping section below. There will be no direct benefits to the study participants; however, knowledge gained from the study could potentially benefit patients in the future.

Study Medication Risks

Severe allergic reactions (rare)

- Swelling of the face, lips, tongue or throat. This may make it difficult to swallow and breath.
- Severe itching of the skin (with raised lumps).
- Nerve damage that may cause changes in sensation or muscle weakness (neuropathy).
- Slowed or stopped breathing or stopped heartbeat.
- Total spinal block
- Uneven heart beat (arrhythmias).

Common

- Low blood pressure (causing dizziness or light-headedness).
- Feeling sick (nausea) or being sick (vomiting).
- Pins and needles.

Uncommon

- Ringing in the ears (tinnitus) or being sensitive to sound.
- Numbness of the tongue or around the mouth.
- Feeling sleepy.
- Shivering.

Risk Mitigation

These risks can occur in any patient undergoing the procedure under anesthesia and are not study specific. All subjects will be observed in a unit accustomed to treating patients recovering from surgery and anesthesia. Customary clinical care will be provided by the patient's treating physician. No standard treatments will be withheld as a result of participation in the study.

Drug Accountability and Subject Compliance

This study will take place within the UAMS hospital's labor and delivery unit. There will be full drug accountability throughout the study. There will be an accountability log, labels for each ampoule marked especially for the study (DPE/Epidural Study). The procedure will be performed by an un-blinded anesthesiologist and the assessments and conduct of surgery will be performed by another anesthesiologist who remains blinded to the patient allocation group. The procedure (DPE or epidural) will be documented within the anesthetic record by the unblinded anesthesiologists. Compliance will be confirmed by comparing the medical chart to the accountability logbook.

Data Handling and Recordkeeping

The Principal Investigator will carefully monitor study procedures to protect the safety of research subjects, the quality of the data and the integrity of the study. All study subject material will be assigned a unique identifying code or number. The key to the code will be kept in a locked file cabinet and password protected Principal Investigator's computer in the Principal Investigator's office. Only Nadir Sharawi, MD will have access to the code and information that identifies the subject in this study. At the conclusion of the study, the data will be permanently deidentified. Deidentified study data will be maintained and ultimately destroyed per UAMS policy.

Data Analysis

The alternative hypothesis is that DPE group will have a faster onset time to achieve loss of sharp sensation at the T6 dermatome compared to the epidural group.

For statistical analysis, the Student's t- test will be used for continuous normally distributed variables and the Mann-Whitney test for nonparametric variables. Linear regression analysis will be used to assess any relationship between the pre epidural extension parameters and the subsequent speed of onset of the block.

<u>Sample Size</u>

The sample size will be calculated for continuous outcome date for a superiority study. We have assumed that an onset time difference of two minutes is the smallest difference that is clinically acceptable, so that a difference of more than two minutes would matter in clinical practice. Approximately 120 mother-infant dyads are required to have a 90% chance of detecting, as significant at the 5% level, an increase in the primary outcome measure from 10 minutes in the DPE group to 12 minutes in the epidural group, assuming a standard deviation of 3 minutes. Therefore 60 mother – infant dyads will be recruited to each arm. Statistical significance will be taken as $P < 0.05$. In total, 140 mother – infant dyads will be recruited to account for any withdrawals or protocol violations.

<u>Randomization</u>

70 pieces of paper will be printed for each group containing (DPE or epidural groups) for a total of 140. Each of the individual pieces of paper will then be placed in a sealed envelope. All envelopes will be shuffled and then numbered 1 – 140.
Patients will be assigned a number 1 – 140 as they are enrolled in the study. The envelope will be obtained and opened by an un-blinded anesthesiologist revealing their randomization group. The un-blinded Anesthesiologist will not be involved in the patient's care or data collection. They will insert the epidural or DPE accordingly. They will not be aware of primary outcome result as this will be documented on a separate data collection form. All members of the patient's care team are blinded to the assignment study drug. The un-blinded anesthesiologist will inform the clinical team which procedure was undertaken if determined to be clinically necessary.

Withdrawal of Participants

Subjects will have the right to withdraw from the study at any point in time and have the right to withdraw any accompanying data. The study has been powered to account for approximately a 15% withdrawal / procedure failure rate.

Stopping the Study

Subject participation in the study will be stopped if either of the following occurs:

- the subject did not achieve sharp sensation lost bilaterally at the T6 dermatomal level (as measured by a blunt plastic neurotip® (Owen Mumford, USA) device) after 18 minutes from administration of 3 ml test dose;
- the subject experiences significant pain that is not relieved by the intraoperative analgesic supplementation described above;
- if general anesthesia must be instituted to the subject.

Ethical Considerations

This study will be conducted in accordance with all applicable government regulations and University of Arkansas for Medical Sciences research policies and procedures. This protocol and any amendments will be submitted and approved by the UAMS Institutional Review Board (IRB) to conduct the study.
The formal consent of each subject, using the IRB-approved consent/HIPAA form, will be obtained before that subject is submitted to any study procedure. All subjects for this study will be provided a consent/HIPAA form describing this study and providing sufficient information in language suitable for subjects to make an informed decision about their participation in this study. The person obtaining consent will thoroughly explain each element of the document and outline the risks and benefits, alternate treatment(s), and requirements of the study. The consent process will take place in a quiet and private room, and subjects may take as much time as needed to make a decision about their participation. Participation privacy will be maintained and questions regarding participation will be answered. No coercion or undue influence will be used in the consent process. This consent/HIPAA form must be signed by the subject and the individual obtaining the consent. A copy of the signed consent/HIPAA will be given to the participant, and the informed consent process will be documented in each subject's research record.

Dissemination of Data

Results of this study may be used for presentations, posters, or publications. The publications will not contain any identifiable information that could be linked to a participant.

References

1. Martin, J.A., et al., *Births: final data for 2012.* Natl Vital Stat Rep, 2013. **62**(9): p. 1-68.
2. Martin, J.A., B.E. Hamilton, and M.J. Osterman, *Births in the United States, 2015.* NCHS Data Brief, 2016(258): p. 1-8.
3. *Practice Guidelines for Obstetric Anesthesia: An Updated Report by the American Society of Anesthesiologists Task Force on Obstetric Anesthesia and the Society for Obstetric Anesthesia and Perinatology.* Anesthesiology, 2016. **124**(2): p. 270-300.
4. Liu, S.S. and S.B. McDonald, *Current issues in spinal anesthesia.* Anesthesiology, 2001. **94**(5): p. 888-906.
5. Bauer, M., et al., *Recent advances in epidural analgesia.* Anesthesiol Res Pract, 2012. **2012**: p. 309219.
6. Wilson, S.H., et al., *Labor Analgesia Onset With Dural Puncture Epidural Versus Traditional Epidural Using a 26-Gauge Whitacre Needle and 0.125% Bupivacaine Bolus: A Randomized Clinical Trial.* Anesth Analg, 2018. **126**(2): p. 545-551.
7. Yadav, P., et al., *Comparison of Dural Puncture Epidural Technique versus Conventional Epidural Technique for Labor Analgesia in Primigravida.* Journal of Obstetric Anaesthesia and Critical Care, 2018. **8**(1): p. 24-28.
8. Cappiello, E., et al., *A randomized trial of dural puncture epidural technique compared with the standard epidural technique for labor analgesia.* Anesth Analg, 2008. **107**(5): p. 1646-51.
9. Gupta, D., A. Srirajakalidindi, and V. Soskin, *Dural puncture epidural analgesia is not superior to continuous labor epidural analgesia.* Middle East J Anaesthesiol, 2013. **22**(3): p. 309-16.
10. Chau, A., et al., *Dural Puncture Epidural Technique Improves Labor Analgesia Quality With Fewer Side Effects Compared With Epidural and Combined Spinal Epidural Techniques: A Randomized Clinical Trial.* Anesth Analg, 2017. **124**(2): p. 560-569.
11. Suzuki, N., et al., *Dural puncture with a 26-gauge spinal needle affects spread of epidural anesthesia.* Anesth Analg, 1996. **82**(5): p. 1040-2.

Appendices

1. Bromage Score
2. Neonatal Apgar Score
3. Pain Visual Analogue Scale (VAS)

Appendix 1

Description of the Bromage score

Grade 1 No motor block

Grade 2 Inability to raise extended leg, able to move knees and feet

Grade 3 Inability to raise extended leg and move knee, able to move feet

Grade 4 Complete motor block of the lower limbs.

Appendix 2

Neonatal Apgar Score

	Score of 0	Score of 1	Score of 2	Component of backronym
**Skin color**	blue or pale all over	blue at extremities body pink (acrocyanosis)	no cyanosis body and extremities pink	**A**ppearance
**Pulse rate**	absent	< 100 beats per minute	> 100 beats per minute	**P**ulse
**Reflex irritability grimace**	no response to stimulation	grimace on suction or aggressive stimulation	cry on stimulation	**G**rimace
**Activity**	none	some flexion	flexed arms and legs that resist extension	**A**ctivity
**Respiratory effort**	absent	weak, irregular, gasping	strong, robust cry	**R**espiration

Appendix 3

Pain Visual Analogue Scale

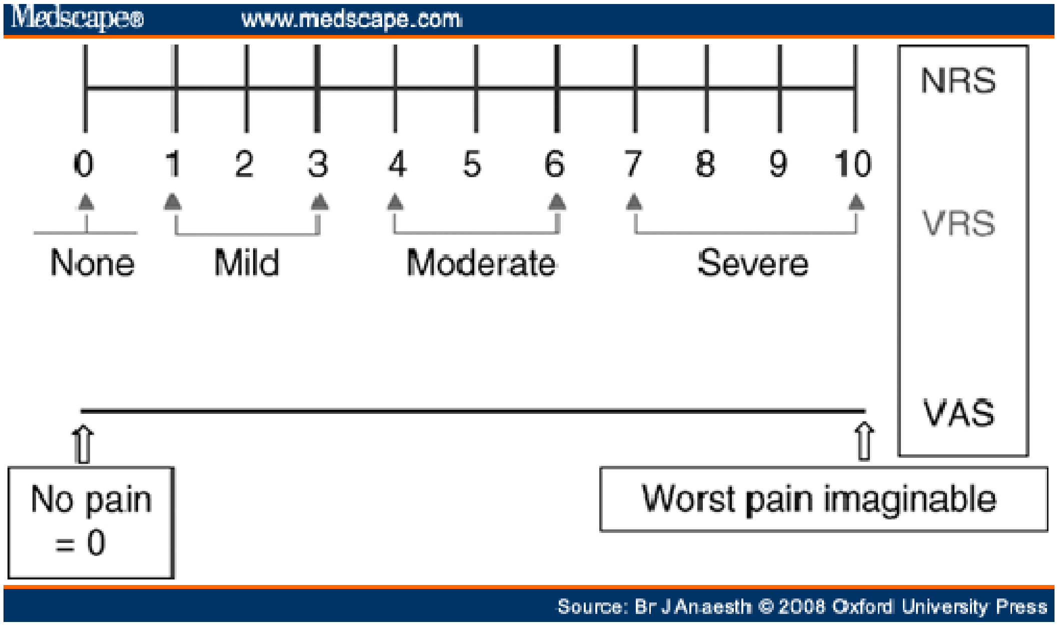

Supplement: Supplement 1. — Trial Protocol [file jamanetwopen-e2326710-s001.pdf]
