# Supplementary material for: Effect of Dural-Puncture Epidural vs Standard Epidural for Epidural Extension on Onset Time of Surgical Anesthesia in Elective Cesarean Delivery: A Randomized Clinical Trial
Source: JAMA Netw Open. 2023 Aug 1;6(8):e2326710. doi: 10.1001/jamanetworkopen.2023.26710 (PMC10394571; doi:10.1001/jamanetworkopen.2023.26710)
Supplement: Supplement 2. — Data Sharing Statement [file jamanetwopen-e2326710-s002.pdf]

Data Sharing Statement

Sharawi. Effect of Dural-Puncture Epidural vs Standard Epidural for Epidural Extension on Onset Time of Surgical Anesthesia in Elective Cesarean Delivery. *JAMA Netw Open*. Published August 01, 2023. doi:10.1001/jamanetworkopen.2023.26710

Data
Data available: No
